# Analysis of Prognostic Factors for Survival after Hepatectomy for Hepatocellular Carcinoma Based on a Bayesian Network

**DOI:** 10.1371/journal.pone.0120805

**Published:** 2015-03-31

**Authors:** Zhi-qiang Cai, Shu-bin Si, Chen Chen, Yaling Zhao, Yong-yi Ma, Lin Wang, Zhi-min Geng

**Affiliations:** 1 Department of Hepatobiliary Surgery, First Affiliated Hospital of Xi’an Jiaotong University, College of Medicine, Xi’an 710061, Shaanxi, China; 2 Department of Industrial Engineering, School of Mechanical Engineering, Northwestern Polytechnical University, Xi’an 710072, Shaanxi, China; 3 Department of Epidemiology and Biostatistics, School of Public Health, Xi’an Jiaotong University Health Science Center, Xi’an 710061, Shaanxi, China; University of Pisa, ITALY

## Abstract

**Background:**

The prognosis of hepatocellular carcinoma (HCC) after hepatectomy involves many factors. Previous studies have evaluated the separate influences of single factors; few have considered the combined influence of various factors. This paper combines the Bayesian network (BN) with importance measures to identify key factors that have significant effects on survival time.

**Methods:**

A dataset of 299 patients with HCC after hepatectomy was studied to establish a BN using a tree-augmented naïve Bayes algorithm that could mine relationships between factors. The composite importance measure was applied to rank the impact of factors on survival time.

**Results:**

124 patients (>10 months) and 77 patients (≤10 months) were correctly classified. The accuracy of BN model was 67.2%. For patients with long survival time (>10 months), the true-positive rate of the model was 83.22% and the false-positive rate was 48.67%. According to the model, the preoperative alpha fetoprotein (AFP) level and postoperative performance of transcatheter arterial chemoembolization (TACE) were independent factors for survival of HCC patients. The grade of preoperative liver function reflected the tendency for postoperative complications. Intraoperative blood loss, tumor size, portal vein tumor thrombosis (PVTT), time of clamping the porta hepatis, tumor number, operative method, and metastasis were dependent variables in survival time prediction. PVTT was considered the most significant for the prognosis of survival time.

**Conclusions:**

Using the BN and importance measures, PVTT was identified as the most significant predictor of survival time for patients with HCC after hepatectomy.

## Introduction

Hepatocellular carcinoma (HCC) is a common malignant tumor with high mortality worldwide, especially in East Asian countries. In China, 360,000 incident cases and 350,000 deaths secondary to HCC occur annually [1]. Although surgical resection and liver transplantation are currently the best curative options to treat HCC, recurrence or metastasis is quite common in patients who have had a resection and the survival rate is 30% to 40% at 5 years postoperatively, with nearly 600,000 people die of HCC each year worldwide [2,3]. However, the contributing factors to the development of HCC are not fully understood, leading to great difficulties in the prediction of survival time and decisions regarding therapy. Therefore, there is a need to identify the key factors that influence the survival of patients with HCC.

Recently, data-based statistical methods have been extensively applied to the analysis of prognostic factors for survival of patients with HCC[4–7]. These studies discussed prognostic factors such as gene expression, tumor size, alpha fetoprotein (AFP) level, and recurrence by statistical analysis of clinical data, which is universally representative of research-oriented thinking. However, these studies only focused on the separate impacts of single factors associated with prognosis and ignored the combined effects of multiple factors. Because survival time prediction involves the influence of many factors whose interactions or mutual influences are not yet clearly understood, an effective modeling method must be proposed to explore and represent the relationships among various factors.

The development of computer technologies has led to the proposal of many artificial intelligence algorithms of data mining. Researchers have analyzed medical data to support the experts in the course of clinical staging, decision-making, and prognosis prediction. Chen et al. [8] proposed a clustering-based approach to the development of prognostic systems for patients with cancer and demonstrated the use of one such method for patients with lung cancer. Nouso et al. [9] analyzed the prognostic factors for and the treatment effect on survival in HCC patients with Child C cirrhosis using Cox proportional hazards regression analysis and propensity score-matched analysis, respectively. Chen et al. [10] adopted an artificial neural network and classification and regression tree as prediction models for liver cancer. Hung et al. [11] proposed an artificial neural network model for the prediction of 5-year mortality after surgical treatment of HCC and compared its performance with that of a logistic regression model, proving that the artificial neural network model was more accurate. Prediction of the prognosis of hepatic resection for HCC is associated with a variety of uncertainties. Although some data mining methods have been developed and applied to survival prediction of patients with HCC, most methods cannot represent variables under uncertainty and ignore the cause–effect relationships between prognostic factors. The Bayesian network (BN) is good at representation of nonlinearity and variable interactions [12], and importance measures are useful tools with which to address uncertainty in model prediction [13]. Therefore, we explored to use a BN for construction of a model for prediction of the prognosis of patients with HCC and to use importance measures to analyze the prognostic factors.

## Methods

### Patients and data collection

In total, 299 original medical records of patients with HCC after hepatectomy were collected from the First Affiliated Hospital of Xi’an Jiaotong University, College of Medicine in China from February 1, 2006 to October 31, 2011.

The dataset of patients was established with 16 columns, including sex, age, HBV history, HCV history, preoperative AFP, preoperative liver function, tumor size, tumor numbers, portal vein tumor thrombosis (PVTT), operative methods, metastasis, time of clamping the porta hepatis (TCPH), intraoperative blood loss, postoperative complication, transcatheter arterial chemoembolization (TACE) and survival time, see Table 1 for the detailed information.

**Table 1:** Standard description of data.

ID	Variables	General description	Values	Type
1	Sex	Female	0	Discrete
		Male	1	
2	Age		16~45,46~59,60~84(years)	Continuous
3	HBV history	N	0	Discrete
		Y	1	
4	HCV history	N	0	Discrete
		Y	1	
5	Preoperative AFP		0~8,8.01~399.99,400~121000(ng/mL)	Continuous
6	Preoperative liver function	Child A	0	Discrete
		Child B	1	
7	Tumor size		0.8~1.9,2~4.9,5~9.9,≥10(cm)	Continuous
8	Tumor number	Single	0	Discrete
		Multi	1	
9	PVTT	N	0	Discrete
		Y	1	
10	Operative method	PAH	0	Discrete
		ANH	1	
11	Cancer metastasis	N	0	Discrete
		Y	1	
12	TCPH	< = 15 mins	0	Discrete
		>15 mins	1	
13	Intraoperative blood loss	< = 400 ml	0	Discrete
		>400 ml	1	
14	Postoperative complication	N	0	Discrete
		Y	1	
15	Postoperative TACE	N	0	Discrete
		Y	1	
16	Survival time		1~10,11~68(months)	Continuous

### Follow-up of patients

The survival time was counted from the operation to the death of patients, to loss to follow up for patients lost to follow-up, or to the end of the follow-up period for patients who were still alive when the study ended in November 2011. The death of patients (41.8%) was confirmed by follow-up surveys. Although 15.4% of patients were lost to follow up, their survival time was more than 10 months which had no influence on the final analysis, so this part of data was efficient. The last part of data (42.8%) was from patients in stable condition, and the observation time was all more than 10 months.

### Bayesian network

A BN is a probability graphical model that represents a set of random variables and their conditional dependencies via a directed acyclic graph. Formally, a BN comprises nodes, edges, and conditional probability. Nodes represent random variables, which may be observable quantities, latent variables, unknown parameters, or hypotheses. Edges represent conditional dependencies, pointing to the child nodes from the parent nodes. Nodes are not connected by edges, indicating that they are conditionally independent of one another. Each node has a conditional probability table (CPT) to quantitatively express the interdependence between nodes. Child nodes take the values of parent nodes as input to generate their CPTs. Some nodes with no parent nodes are called root nodes, and their CPTs are determined by experience and knowledge.

Through the application of Bayes theory, data mining tasks such as prediction, classification, and causal analysis can be performed with the support of a BN model. Assuming that all variables are conditionally independent in a given class, the naïve Bayes classifier learns the conditional probability of each variable from training data and applies the Bayes rule to compute the probability of a class given a particular variable. The above assumption is usually unrealistic for attribute variables, which are dependent in many cases; e.g., physical indicators in disease diagnosis. Friedman et al. [14] relaxed the assumption of conditional independence and proposed a tree-augmented naïve Bayes (TAN) method, which outperforms naïve Bayes. Cai et al. [15] brought forward a new conditional BN model to identify the product failure rate grade under diverse configuration and operation conditions. Si et al. [16] established a breast cancer diagnosis model to identify tumor markers based on BN using a real-world database.

### Prognostic model based on Bayesian networks

Because of its perfect ability to present the nonlinearity and cause–effect relationships, the BN has become popular among researchers as a useful data-mining tool. Easily understood BN models can be readily established with the assistance of professional software. Because the data format in the original dataset was uniform and not easily recognized by computers, standardization of the description of the data was required before modeling.

Additionally, the BN only operates well with a variable of fewer than six discrete states; continuous prognostic factors should be first transformed into discrete intervals according to the features of data concentration. In this study, age was divided into three intervals of 16~45, 46~59, and 60~84 years. According to the significance of the AFP concentration at different stages of prognosis, the AFP concentration was divided into three intervals of 0~8, 8.01~399.99, and 400~121,000 ng/mL. Tumor size was divided into four intervals of <2, 2~4.9, 5~9.9, ≥ 10 cm based on medical definitions. Survival time was divided into two intervals of ≤ 10 and >10 months according to the equal frequency principle.

**Fig 1:**
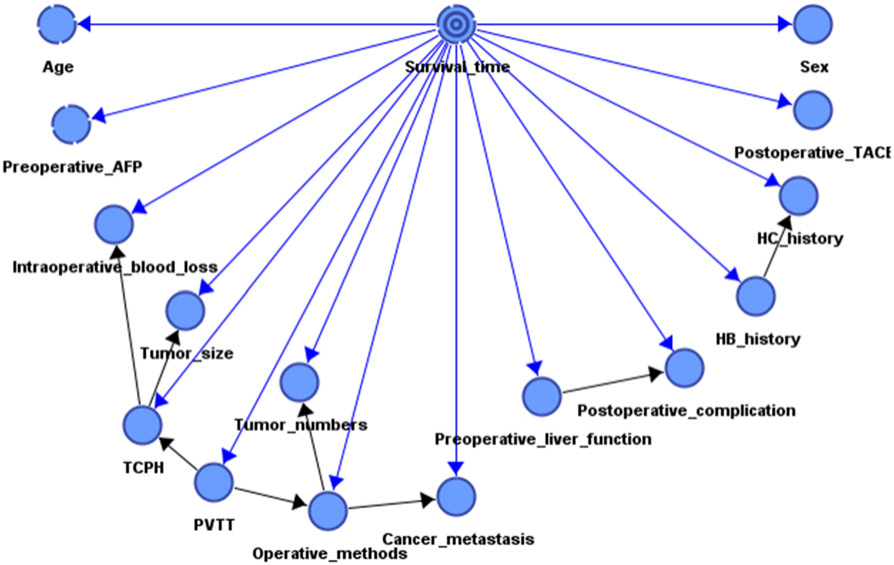
BN model for prognostic factors.

According to the concepts and constraints of the BN classifier, the prognostic model was established from the dataset of patients. The survival time was set as the target variable to be predicted, and other factors were attribute variables which affect the state of the target variable. Then, we used the TAN algorithm to learn the structure and network parameters from the dataset and thus elucidate the cause–effect relationship between these attribute variables, see Fig. 1.

### Confusion matrix, receiver operating characteristics(ROC) curve, and Lift Curve

Before information was obtained from the model, we verified the effectiveness of the model to determine whether it reflected the actual relationships among variables. A confusion matrix is a basic tool with which to evaluate the credibility of a classification model. A binary classification problem is illustrated as an example in Table 2. The columns correspond to the actual cases, and the rows correspond to the outcomes of the classifier. The four categories represent cases that a classifier may encounter. True positive (TP) and true negative (TN) indicate the number of instances classified correctly. False positive (FP) indicates the number of instances misclassified as positive, and false negative (FN) indicates the number of instances misclassified as negative.

**Table 2:** Confusion matrix for a binary classification problem.

		Actual	
		Positive	Negative
Predicted	Positive	True Positive (TP)	False Positive(FP)
	Negative	False Negative(FN)	True Negative(TN)

Additional evaluation indexes were derived from these four basic indexes. Model reliability is defined as the proportion of cases that were correctly predicted to be positive or negative cases. The true-positive rate (TPR) is the proportion of positive cases that were correctly identified and is calculated as *TP / (TP + FN)*. The false-positive rate (FPR) is defined as the proportion of positive cases that were misclassified and is calculated as *FP / (TN + FP)*. Model accuracy is determined by the proportion of predictions that are correct and is calculated as shown by using the following (Equation 1).

$$accuracy=\frac{TP+TN}{TP+FP+TN+FN}.$$ (1)

The accuracy as determined by (Equation 1) may not be an adequate performance measure when the number of negative cases is much greater than the number of positive cases, as when the proportion of positive and negative samples is 1:99. As long as the classification model judges all samples as negative, its accuracy reaches 99%. However, the evaluation index has no reference sense. Additionally, Bayesian classifiers do not simply operate by judging samples as 0 or 1 when determining the classification, but instead operate by the probability of a classification. For these classifiers, different classification and classifier evaluation indexes are obtained when taking different thresholds. Therefore, the receiver operating characteristics (ROC) curve and the area under the curve were introduced to measure the overall credibility of the classification model.

The ROC curve was originally derived from signal detection theory in the 1970s, which describes the relative changes in the TPR–FPR ratio of the classification confusion matrix. The basic principle is that the target variable distinguishes the change in the threshold to obtain pairs of the TPR and FPR. The vertical axis displays the TPR, and the horizontal axis displays the FPR. If the curve almost coincides with the diagonal line (the so-called line of no discrimination) from the left bottom to the top right corners, it shows that the attribute variables have poor judgment value for the target variable. The further the curve is away from the line (i.e., the larger the area under the curve is), the better the judgment value the attribute variables have on the target value.

### Importance measures

Systems are becoming more complex with modern technology and higher reliability requirements. Therefore, identification of the most problematic components may be difficult. Importance measures were proposed to quantify the contributions of individual components to the system performance or the impact of component performance reduction on the system. Birnbaum [17] was the first to use this concept to represent the impact of a component state on the reliability of a system. The Fussell–Vesely (F-V) importance [18] was built to determine which component state has the highest probability to cause failure of a system when the component failure is uncertain. Because the F-V importance can only deal with binary systems, Ramirez-Marquez and Coit [19] developed a multistate system. Importance measures are now widely used to identify key factors of a system.

Because the variables studied herein had more than two states, a composite importance measure was adopted to calculate the importance of factors influencing the survival time of patients with HCC. F-V importance was calculated as follows:

$$I{\left(FV\right)}_{V_{i}^{j}}^{S}=\frac{P(S=0)-P(S=0|V_{i}=j)}{P(S=0)}.$$ (2)

where the survival time *S* was influenced by *n* variables described as *{V*_*1*_, *V*_*2*_, *…*, *V*_*i*_, *…*, *V*_*n*_ *}*, and the variable *V*_*i*_ had *m*_*i*_ *+* 1 candidate states described as {0,1, *…*, *i*, *…*, *m*_*i*_}. Here, the influence of factors on survival time of <10 months was studied.

In the field of reliability engineering, importance measures describe changes in a system when the state of a component changes with respect to a normal one. Because the probability of the normal state was larger than the probability of any other failure state and the structure function of the system was nondecreasing, the importance measure was generally positive. However, there is not so-called normal state in uncertainty; namely, we could not determine which state of a variable should have a larger probability. Therefore, the survival time might increase or decrease when the variable state changes, and negative importance values may be obtained. The composite importance measure generalization for F-V importance was expressed as follows:

$$MFV_{i}=\frac{1}{m_{i}-1}\sum_{j=1}^{m_{i}}abs(I{\left(FV\right)}_{V_{i}^{j}}^{S}).$$ (3)

### Statistical analysis

SPSS 13.0 for Windows (SPSS Inc., Chicago, IL, USA) was used for statistical analyses. Bayeslab (Bayesia Limited Company, France) was used for the establishment of Bayesian network. Continuous variables were expressed as the median (quartile range). Survival rates were calculated with the Kaplan Meier method and compared using the log-rank test. Pearson correlation coefficient was used for the test of the relationships among attribute variables. All statistical tests were two-tailed, and statisitical significance was set at P <0.05.

### Ethics Statement

This study was approved by the Ethics Committee of the First Affiliated Hospital of Xi'an Jiaotong University, College of Medicine. All patients gave written informed consent to participate. The parents wrote the informed consent on behalf of the patients whose age<18. The ethics committee approved this consent procedure. The data did not contain any information that could identify the patients.

## Results

### General characteristics of the study population

Of these patients, 241 (80.6%) were male and 51 (19.4%) were female. The median age at the time of surgery was 52 (quartile range, 43~61) years. 229 (76.6%) patients were positive for hepatitis B surface antigen and 16 (5.4%) were positive for hepatitis C antibody. The proportions of patients with a tumor size of ≥10, 5~9.9, 2~4.9 and <2 cm were 19.4%, 30.1%, 48.5% and 2%, respectively. Metastatic tumor was detected in 46 (15.4%) patients, and 50 (16.7%) patients had more than one tumor. PVTT was observed in 25 (8.4%) patients. The preoperative liver function of 245 (81.9%) and 54 (18.1%) patients was Child–Pugh grade A and B, respectively. The median preoperative AFP level was 181.4 (quartile range, 13~1519) ng/mL. The lesions of 260 (87.0%) patients were resected by anatomical hepatectomy (ANH), in which the lesion was resected completely, and those of 39 (13.0%) patients underwent palliative hepatectomy (PAH), which was performed for the primary tumor combined with intrahepatic or extrahepatic metastasis, PVTT, or the margin had residual tumor. Because the blood loss (≥400 mL, 52.2%) and TCPH (≥15 min, 52.5%) during surgery were important indicators of the prognosis, they were taken into consideration. After hepatectomy, 138 (46.2%) patients developed postoperative complications. Ascites and pleural effusion were minor complications (77.5%), and the major complications (22.5%) included abdominal infection(13, 9.42%), bleeding (9, 6.52%), bile leakage (3, 2.17%), pulmonary infection (2, 1.45%), hepatic encephalopathy (1, 0.72%), jaundice (1, 0.72%), stress ulcer (1, 0.72%), and hydropneumothorax (1, 0.72%). TACE was conducted on 139 (46.5%) patients, and the majority of TACE (81.3%) was preventive intervention.

### Survival analysis

**Fig 2:**
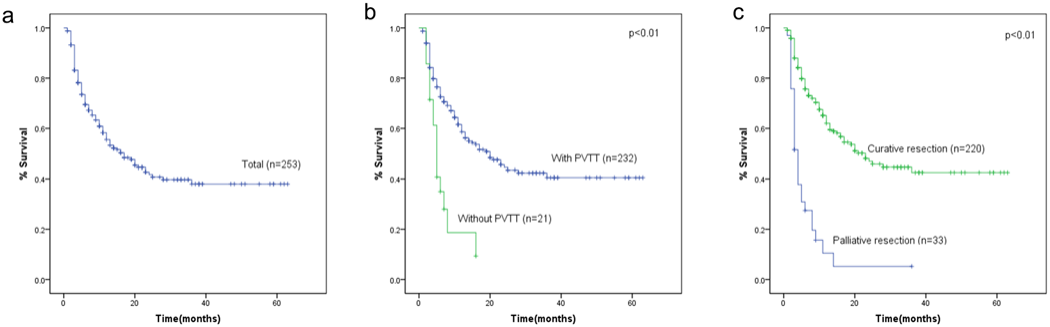
(A) Overall survival of HCC patients after hepatectomy. (B) Survival between HCC with PVTT and without PVTT. (C) Survival between HCC with curative resection and palliative resection.

The survival curve is shown in Fig. 2. Overall, the 1-year and 3- year survival rate was 56% and 38%, respectively, and the median survival time was 17.0 (95%CI:11.4~22.6) months. The median survival time of HCC with PVTT and without PVTT was 5.0 (95%CI:3.9~6.1) months and 20.0 (95%CI:13.1~26.9) months respectively and the difference in survival time between those two groups was statistically significant (P<0.01). The median survival time of HCC with curative resection and palliative resection was 23.0 (95%CI:15.2~30.8) months and 4.0 (95%CI:3.1~4.9) months respectively, and the difference was statistically significant (P<0.01).

### Assessment of model efficacy

Values of the attribute variables in the dataset were input into the established BN model, and the survival times obtained by reasoning were compared with those obtained by the original records. Based on the definition of confusion matrix evaluation indexes, the reliability and accuracy of the reasoning results were calculated (Table 3). The actual number of patients (>10 months) was 149, of whom 124 were correctly classified; thus, the corresponding accuracy was 83.22%. The number of patients (>10 months) obtained by the model was 197, of whom 124 had a survival time of >10 months; thus, the reliability was 62.94%. This was the predicted rate of correct classification. In total, 124 patients (>10 months) and 77 patients (≤10 months) were correctly classified, and the model accuracy was thus 67.2% (calculated as in (Equation 1)). Because it was difficult for a classifier to identify all cases, especially in cases involving multiple influencing factors, the accuracy of the model was acceptable.

**Table 3:** Confusion matrix and reliability and accuracy of the BN model of prognosis.

	Survival time(n)	< = 10m (n = 150)	>10m (n = 149)
Confusion matrix(n)	< = 10m (102)	77	25
	>10m (197)	73	124
Reliability(%)	< = 10m (102)	75.49%	24.51%
	>10m (197)	37.06%	62.94%
Accuracy(%)	< = 10m (102)	51.33%	16.78%
	>10m (197)	48.67%	83.22%

**Fig 3:**
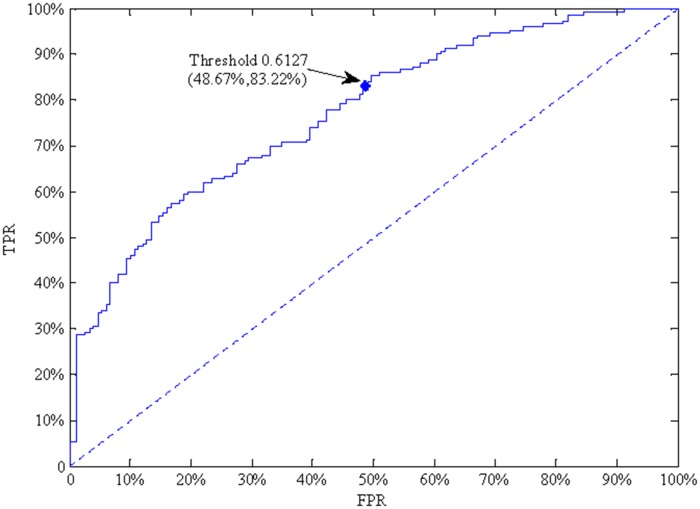
ROC curve of a survival time of >10 months.

Because the BN determined a classification according to probability, the probability could be taken as the discrimination threshold. A patient was classified as having a long survival time (>10 months) when the probability was more than the threshold; otherwise, the patient was classified as having a short survival time (≤10 months). The threshold was set at 0.6127 in the present study so that the model achieved the highest accuracy. For patients with long survival times, the TPR of the model was 83.22% and the FPR was 48.67% at the threshold (Fig. 3). As the threshold varied from 0 to 1, the corresponding FPR and TPR formed the ROC curve (Fig. 3). Thus, we obtained a higher TPR with a given FPR, meaning that we obtained higher prediction accuracy with lower risk.

### Cause-effect relationships among attribute variables

Among the factors studied, the preoperative AFP level and postoperative performance of TACE were independent factors for survival of patients with HCC patients. The statistical properties of hepatitis B and C showed a weak linear correlation (Pearson correlation coefficient [PCC], 0.2192). The grade of preoperative liver function reflected the tendency for postoperative complications to some extent (PCC, 0.2106). For example, 215 (72%) patients whose preoperative liver function was Child-Pugh class B developed complications after hepatectomy. Intraoperative blood loss, tumor size, PVTT, TCPH, tumor number, operative methods, and metastasis were dependent variables in the prediction of survival time.

### Prognostic factors ranked by importance

The importance of various factors was analyzed based on the established BN prognostic model. First, we obtained the prior probability distribution of each factor, as shown in Table 4. The prior probability of survival time was {*p*(*S* = 0) = 0.5017, *p*(*S* = 1) = 0.4983}, and the prognostic factors that were attribute variables were described as {*p*(*V* = 0), *p*(*V* = 1),…}. Next, every state of the attribute variables was modified and the posterior probability distribution of a survival time of <10 months was calculated. The posterior probability was determined by {*p*(*S* = 0|*V* = 0), *p*(*S* = 0|*V* = 1),…}. Finally, the importance measure of each variable was calculated. The results were shown in Table 4.

**Table 4:** Importance of prognostic factors in survival time ranking.

Prognostic factors	State	Priori probability p(Vi)	Posterior probability p(S = 0|Vi = j)	FVi	MFVi	Rank 1	Person correlation	Rank 2
Sex	0	0.194	0.5172	-0.0309	0.0385	14	0.0153	14
	1	0.806	0.4979	0.0076				
Age	16–45	0.3311	0.5051	-0.0068	0.0068	15	0.0041	15
	45–60	0.408	0.5	0.0034				
	60–84	0.2609	0.5	0.0034				
HBV history	0	0.2341	0.5429	-0.0821	0.1072	13	0.0455	12
	1	0.7659	0.4891	0.0251				
HCV history	0	0.9465	0.5159	-0.0283	0.53	6	0.1197	9
	1	0.0535	0.25	0.5017				
Preoperative AFP	0–8	0.184	0.4	0.2027	0.1468	12	-0.0251	13
	8–400	0.4147	0.5323	-0.061				
	400–121000	0.4013	0.5167	-0.0299				
Preoperative liver function	0	0.8194	0.4816	0.0401	0.2212	11	-0.0854	10
	1	0.1806	0.5926	-0.1812				
Tumor size	0	0.0201	0.1667	0.6677	0.4663	7	-0.2044	5
	1	0.4849	0.4	0.2027				
	2	0.301	0.5444	-0.0851				
	3	0.194	0.7241	-0.4433				
Tumor number	0	0.8328	0.4498	0.1034	0.6183	4	-0.2315	3
	1	0.1672	0.76	-0.5148				
PVTT	0	0.9164	0.4708	0.0616	0.7359	1	-0.2628	1
	1	0.0836	0.84	-0.6743				
Operative	0	0.1304	0.8205	-0.6354	0.7309	2	0.247	2
Method	1	0.8696	0.4538	0.0955				
Cancer metastasis	0	0.8462	0.4585	0.0861	0.5593	5	-0.2025	6
	1	0.1538	0.7391	-0.4732				
TCPH	0	0.4749	0.3803	0.242	0.4608	3	-0.2309	4
	1	0.5251	0.6115	-0.2189				
Intraoperative blood loss	0	0.4783	0.4196	0.1636	0.3135	9	-0.1572	8
	1	0.5217	0.5769	-0.1499				
Postoperative complication	0	0.5385	0.4658	0.0716	0.1549	10	-0.0774	11
	1	0.4615	0.5435	-0.0833				
Postoperative TACE	0	0.5351	0.5937	-0.1834	0.3947	8	0.1976	7
	1	0.4649	0.3957	0.2113				

The PCC between prognostic factors and survival time were calculated and ranked according to their absolute values to allow for comparison by rank of importance. Both ranks showed that PVTT was the most significant factor for the prognosis of survival time with respect to the mentioned factors. The anatomical hepatectomy (ANH) was also an important prognostic factor. According to these results, neither age nor sex had a significant influence on survival time.

## Discussion

The purpose of this study was to introduce a method with which to combine BN with importance theory and thus identify key factors under uncertainty. Based on Bayesian theory and data mining technology, a BN model was established for survival time prediction with the dataset gathered from the First Affiliated Hospital of Medical College of Xi’an Jiaotong University in China. As a theoretical model, the BN model can not only discover the hidden relationships among factors, but also express the relationships in an understandable way and has been widely used in medicine. In a study by Aguiar-Pulido et al. [20], a structure–disease relationship model was created to discover new proteins associated with cancer, and the model showed excellent predictive ability (90.92%). In total, 124 patients (>10 months) and 77 patients (≤10 months) were correctly classified, and the model accuracy was thus 67.2%. It was difficult for a classifier to identify all cases, especially in cases involving multiple influencing factors, the accuracy of the model was acceptable. For patients with long survival time, the TPR of the model was 83.22% and the FPR was 48.67% at the discrimination threshold set at 0.6127. Thus, we obtained a higher TPR with a given FPR, suggesting that we obtained higher prediction accuracy with lower risk.

Static and dynamic characteristics of the model were verified, indicating that the model embodied the information of the dataset. According to the model, the PVTT, tumor number, metastasis, and operative method were dependent variables in the prediction of survival time. Many previous studies have revealed PVTT is one of the most important prognostic factors [21]. This was also confirmed in the present study, and most HCCs with PVTT were technically unresectable and unsuitable for other curative therapies [22]. The median survival time of HCC with PVTT and without PVTT was 5.0 months and 20.0 months respectively, there was a significant difference in survival time between two groups (P<0.01). The median survival time of HCC with curative resection and palliative resection was 23.0 months and 4.0 months respectively, and there was a significant difference in survival time between two groups (P<0.01). Based on the established model, the posterior probability of survival time can be calculated for patients with PVTT when treated with different surgical methods, as shown in Table 5. The probability of survival time of >10 months was 7.14% with PAH resection and 27.27% with ANH resection, suggesting that the surgical method influences the survival time and curative resection could prolong the survival time of patients with HCC and PVTT. Additionally, surgical factors such as intraoperative blood loss and TCPH influenced the survival time of patients with concurrent HCC and PVTT.

**Table 5:** The posterior probability of survival time for patients with PVTT.

		Metastasis	Blood loss	TCPH	Tumor number	Survival time
PVTT = 1	PAH	P(1) = 51.53%	P(1) = 68.87%	P(1) = 84.95%	P(1) = 48.47%	P(>10) = 7.14%
	ANH	P(1) = 12.44%	P(1) = 66.62%	P(1) = 82.79%	P(1) = 15.48%	P(>10) = 27.27%

A comparison was made between the ranks of factors obtained by importance measures and PCC with respect to survival time (Table 4). The result was almost the same, showing that the rank of importance was credible. Both ranks showed that PVTT was the most significant factor among all studied variables, which is consistent with the results of previous studies [21–23]. Patients with HCC complicated by portal PVTT have an extremely poor prognosis. The median survival time of patients with concurrent HCC and PVTT was 5.6 months in the dataset, including all patients treated with various operative methods, while TACE was helpful to prolong the survival time of select patients. A recent study showed that liver resection is justified in select patients with PVTT located in the segmental or sectoral branches of the portal vein [24], and combined treatment involving radiation for PVTT and TACE for liver tumors achieved a high response rate [23].

Because the BN model reflected the ability of the cause–effect relationship of these variables to predict the probability of survival time, it can be used to quantitatively measure the influence of a factor on survival time and provide guidance for the determination of the optimal treatment. A patient with PVTT and multiple lesions could be used as an example to explain how to optimize the treatment using the model. If the lesions of the patient were resected through PAH and ANH, respectively, the posterior probability of survival time >10 months was 4.21% and 12.41%, respectively. If some positive measures were taken, such as adopting TACE treatment, choosing an operative method with less blood loss, or shortening the TCPH, the aforementioned probability could be increased to 11.15% and 28.8%, respectively, as shown in Table 6. Using this model and the relative importance ranking of factors, we were able to test the efficacy of various treatment options through simulation to make the better treatment choice.

**Table 6:** Simulation to prolong the survival time of patients with PVTT.

					Operative methods	Survival time
PVTT = 1	Tumor Number = 1	TACE = 1	Blood loss = 0	TCPH = 1	PAH	P(>10) = 11.15%
					ANH	P(>10) = 28.8%

In summary, we combine the Bayesian network (BN) with importance theory to identify key factors that have a combined effect on survival after hepatectomy for HCC. Our data suggests that Bayesian network is an effective tool for medical data mining and importance measures can be applied in medicine to analyze the influence of variables related to a target. PVTT is a significant predicator of survival time for HCC patients.

However, sufficient data obtained from patients can help achieve a high predictive accuracy [25], which was unsatisfactory in this study. There were 15 attribute variables in the model, a dataset of 299 records could be used to explore the cause–effect relationship among them; and this number was too small to accurately present the relationship. Additional clinical records of patients with HCC should be collected for future research.

## Supporting Information

S1 Table Dataset of 299 patients with HCC underwent hepatectomy. (XLS)
